# Association of Adipose Tissue Insulin Resistance With Risk of Diabetes Incidence in Middle-aged Japanese Workers According to BMI States: 17 Years of Follow-up of the Aichi Worker’s Cohort Study

**DOI:** 10.2188/jea.JE20250025

**Published:** 2026-01-05

**Authors:** Tahmina Akter, Zean Song, Midori Takada, Mohammad Hassan Hamrah, Shuang Wang, Baruck Tegegn Endale, Shalini Enon Perera Paththamesthrige, May Thet Khine, Masaaki Matsunaga, Atsuhiko Ota, Koji Tamakoshi, Hiroshi Yatsuya

**Affiliations:** 1 Department of Public Health and Health Systems, Nagoya University Graduate School of Medicine, Nagoya, Japan; 2 Department of Public Health, Fujita Health University School of Medicine, Aichi, Japan; 3 Department of Nursing, Nagoya University School of Health Sciences, Nagoya, Japan

**Keywords:** type 2 diabetes mellitus, Adipo-IR, overweight, Japanese, BMI

## Abstract

**Background:**

Insulin resistance in adipocytes, manifested as high basal circulating free fatty acid (FFA) is thought to contribute to the development of type 2 diabetes mellitus (T2DM). However, the association between adipocyte insulin resistance (Adipo-IR) index and T2DM has rarely been explored in prospective studies. We examined this association in a middle-aged Japanese workers’ cohort. Since the association may differ according to the degree of overall adiposity, the analysis was stratified by the presence of overweight/obesity defined with body mass index (BMI).

**Methods:**

A total of 3,257 subjects (men 2501, women 756) aged 35–66 years were followed-up for up to 17 years. T2DM incidence was defined as fasting blood glucose level ≥126 mg/dL, glycated hemoglobin level ≥6.5%, or self-reported initiation of glucose-lowering medications. Adipo-IR was calculated as the product of FFA (mmol/L) and insulin (pmol/L) obtained from baseline fasting blood samples and divided into sex- and BMI category (<25 or ≥25 kg/m^2^)-specific tertiles. Cox-proportional hazards model was used to estimate hazard ratios (HRs) and 95% confidence intervals (CIs) adjusted for age, BMI, smoking status, physical activity, drinking habit, and family history of diabetes.

**Results:**

During a median of 14.6 years of follow-up, 365 developed T2DM. Compared with the lowest tertile, T2DM risk was significantly increased among the highest tertile category in overweight/obese men (HR 2.94; 95% CI, 1.76–4.90) and women (HR 4.24; 95% CI, 1.08–16.61).

**Conclusion:**

Adipo-IR was positively associated with T2DM risk in overweight/obese men and women.

## INTRODUCTION

Adipose tissue is not solely an energy reservoir; it also functions as a dynamic endocrine organ that synthesizes and secretes hormones and other bioactive molecules collectively known as adipocytokines. It is also the primary target organ of insulin action, which regulates the release of free fatty acids (FFA) and glycerol into the circulation by inhibiting lipolysis.^1^^,^^2^ There was indeed an inverse relationship between insulin-sensitivity and plasma FFA concentration.^3^ However, under the state of insulin resistance of adipocyte, this dose-response curve shifted to the right; in other words, at the same insulin levels, lipolysis would not be suppressed as much and circulating FFA levels would become higher.^4^ In such states, chronically higher levels of plasma FFA would further defect the insulin signaling pathway, which may cause lipid accumulation in ectopic sites such as liver, pancreas, and skeletal muscle.^4^^–^^6^

The suppressed antilipolytic effect of insulin in adipose tissue could be measured by multiplying the fasting FFA concentration and the fasting insulin concentration, known as adipose tissue insulin resistance (Adipo-IR).^7^ Cross-sectional studies suggested that Adipo-IR was higher among overweight or obese people compared to normal-weight individuals and it gradually increased from normal to impaired glucose tolerance (IGT) and to type 2 diabetes mellitus (T2DM).^8^^–^^10^ Longitudinal studies also reported that elevated Adipo-IR was associated with increased risk of incident dysglycemia^11^ and diabetes.^12^ However, these studies had a smaller sample size or focused on high risk groups such as individuals with IGT.

Although two prospective studies on individuals at high risk of T2DM examined the association of Adipo-IR with subsequent development of T2DM, no previous prospective study has investigated the association among apparently healthy individuals or by stratifying by body mass index (BMI) category. The present study, with a large sample size, aimed to explore the association between baseline Adipo-IR and incident T2DM, stratified by BMI category in middle-aged Japanese men and women.

## METHODS

### Study population

The baseline data was obtained in 2002 from the Aichi Worker’s Cohort Study, which is an ongoing, prospective epidemiological study of non-communicable diseases (*n* = 6,648).^13^^,^^14^ The participants comprised of 35 to 66-year-old civil servants in Aichi Prefecture, which mainly includes urban and suburban areas located in central Japan. A self-administered questionnaire survey was carried out at baseline, and the present study included those who responded to the survey, gave written consent about utilizing their annual worksite health checkup data, medical history and agreed to provide residual blood samples used for health checkups. Of the 4,213 participants who donated serum samples for health check-ups, we excluded subjects with (1) a history of diabetes at baseline (fasting blood glucose level (FBG) ≥126 mg/dL or glycated hemoglobin level (HbA1c) ≥6.5% or self-reported usage of glucose-lowering medication including insulin^15^ (*n* = 396), (2) lost to follow up (*n* = 238), (3) missing FFA level at baseline (*n* = 242), and (4) missing triglycerides (TG) (*n* = 48). Also, we excluded implausibly high values of Adipo-IR, defined as those at 99^th^ percentile or higher (*n* = 32), leaving, 3,257 (age, 35–66 years; 2,501 men, 756 women) participants for the current analysis (eFigure 1).

Participants were followed up through the workplace healthcare department until their retirement. Although the standard retirement age was 60 years, some participants were re-employed and remained under follow-up via the healthcare department. Retirees not re-employed were followed up via postal mail with prior consent; otherwise, they were censored at retirement. The study protocol was approved by the Ethics Review Committee of Nagoya University Graduate School of Medicine (approval number: 2007–0504).

### Anthropometric measurements and biochemical analysis

Height and weight were obtained without wearing shoes but in regular indoor clothing to the nearest 0.1 cm and nearest 0.1 kg, respectively. BMI was calculated as weight in kilograms divided by height in meters squared. Participants were divided into two subgroups; normal weight (BMI <25.0 kg/m^2^) and overweight/obesity (BMI ≥25.0 kg/m^2^).^16^ Venous blood samples were taken after 8 hours or more, or overnight fasting. The serum samples were frozen at −80°C until the biochemical assay in a commercial laboratory. FFA and serum insulin levels were measured using an enzymatic method and solid-phase radioimmunoassay (RIABEAD II; Dinabot Co., Ltd., Chiba, Japan), respectively. TG level was determined enzymatically. Serum adiponectin was measured using ELISA (Otsuka Pharmaceutical Co., Ltd., Japan). Solid-phase radioimmunoassay (RIABEAD II; Dinabot Co., Ltd., Chiba, Japan) was used to assess fasting serum blood insulin. Fasting blood glucose level (FBG) was enzymatically determined using the hexokinase method. Alanine transaminase (ALT), gamma-glutamyl transpeptidase (GGT), and HbA1c were also measured using the consensus method of the Japan Society of Clinical Chemistry. Adipo-IR was calculated as FFA (mmol/L) × insulin (pmol/L)^7^ and higher Adipo-IR values indicate greater adipose tissue insulin resistance, reflecting reduced suppression of lipolysis by insulin. Additionally, systemic insulin resistance was measured using homeostatic model assessment for insulin resistance (HOMA-IR) index [(blood glucose (mg/dL) × insulin (mUl/L))/405].

### Follow-up and ascertainment of T2DM incidence

The cohort was followed up through March 2019. The incidence of T2DM was identified in two ways. First, we defined incidence as the first occurrence of FBG ≥126 mg/dL or if available HbA1c ≥6.5% (National Glycohemoglobin Standardization Program) in the annual health check-up at the work site. Second, it was self-reported approximately biennially from 2006 to 2019 using a questionnaire. The majority of cases were ascertained through the annual health check-up (86%). Participants who reported a medical history of T2DM were asked to provide contact information for their current or former physicians to verify the validity of the self-report. Approximately half of the self-reported cases were confirmed by physicians. Most of the unconfirmed cases were due to our inability to reach the physicians, primarily because of lack of consent or insufficient contact information. According to our previous study in the Aichi Workers’ Cohort Study, the validity of T2DM self-report was reported to be 95%.^17^

### Other variables

Smoking status was categorized into current, past, and never. Physical activity was assessed using self-report and defined as ≥60 min per day for >12 days per month. Drinking habits were classified as overdrinking or not, with overdrinking defined as ethanol consumption of ≥40 g/day for men and ≥20 g/day for women. Missing values in smoking status and physical activity were coded as missing and included in the analyses. Family history of diabetes was self-reported and defined as the presence of diabetes in the first-degree relatives of the participants. Medication use for hypertension and hyperlipidemia was self-reported.

### Statistical analysis

Mechanistic diagram of Adipo-IR leading to T2DM, illustrating confounding and mediating pathways in statistical analysis was shown in eFigure 2.

All the analyses were conducted in men and women separately. Also, these analyses were stratified by the presence of overweight. Adipo-IR was divided into three-groups (denoted as T1, T2, and T3) according to the tertile cutoff values determined in each sex- and BMI-category stratum. Means and percentages were calculated across Adipo-IR tertiles and tested using analysis of variance and chi-squared test, respectively, unless otherwise indicated. Medians and interquartile ranges were presented for TG, adiponectin, ALT, GGT, and insulin and tested using Kruskal-Wallis test. In addition, correlations of Adipo-IR with other continuous variables were examined using Pearson correlation coefficients. Hazard ratios (HRs) and the 95% confidence intervals [CIs] of Adipo-IR tertiles taking the lowest tertile (T1) as the reference for T2DM incidence were estimated using Cox models adjusted successively for potential confounding factors. The first model was age-adjusted. Multivariable model included age (continuous), BMI (continuous), smoking status,^18^ physical activity, drinking habit, and family history of diabetes^19^ in addition to Adipo-IR. In addition to the Adipo-IR tertile analyses, sex-specific one standard deviation (SD) increments of Adipo-IR were examined in the Cox model as continuous analyses. Proportional hazard assumptions were tested using the Schoenfeld residuals. Effect modification by BMI category in the association of Adipo-IR with T2DM incidence was evaluated by including an interaction term between Adipo-IR and BMI category using the Wald test. Similarly, the interaction between Adipo-IR and sex among overweight/obese participants was also tested using the Wald test. We had conducted several sensitivity analyses to demonstrate that variations in inclusion/exclusion criteria and variable definition did not materially affect the main findings. The first sensitivity analysis was performed excluding participants aged 60 years or older at baseline and censoring those who reached age 60 during follow-up. This was based on the fact that the usual retirement age at the worksite was 60 years, and follow-up procedures after retirement differed from those before retirement. Second, we carried out analyses related to the handling of outliers in Adipo-IR values. Specifically, we compared results without excluding outliers (defined as values at or above the 99^th^ percentile) (*n* = 3,289) and more stringent exclusion of potential outliers (defined as values at or above the 95^th^ percentile) (*n* = 3,125). Third, instead of using sex- and BMI-category specific cutoff values to define Adipo-IR tertiles, we conducted analyses using sex-specific tertiles. Fourth, we also performed analyses using natural log-transformed continuous Adipo-IR values to assess the impact of transformation, given the presence of possible extreme values even after outlier exclusion. All the statistical analyses were conducted with SPSS version 29 (IBM Corp, Armonk, NY, USA). A two-sided *P*-value of <0.05 was considered statistically significant.

## RESULTS

The mean age did not differ across Adipo-IR tertiles in normal weight as well as in either overweight/obese men (Table 1) and women (Table 2). In contrast, the mean BMI was significantly different across Adipo-IR tertiles in all sex- and BMI-category specific strata, except for overweight/obese women. Correlation coefficients between Adipo-IR and TG were 0.40 and 0.45 in normal weight and overweight/obese men (both *P* < 0.01) (eTable 1). They were 0.30 and 0.38 in normal weight and overweight/obese women (both *P* < 0.01).

**Table 1.** Baseline characteristics according to Adipo-IR tertiles stratified by BMI-category in men, Aichi Worker’s Cohort study, 2002 (*n* = 2,501)

	Normal Weight				Overweight/Obesity			
	
	Adipo-IR tertile^a^			*P*	Adipo-IR tertile^a^			*P*
	
	T1	T2	T3		T1	T2	T3	
Number of subjects	642	623	651		193	193	199	
Median Adipo-IR (range)	13.2 (3.0–18.6)	25.2 (18.7–34.2)	52.1 (34.3–227.7)		22.4 (6.9–31.7)	42.2 (31.8–55.8)	80.0 (56.0–225.3)	
Age, years	47.6 (7.0)	47.7 (7.1)	47.9 (6.8)	0.68	48.5 (6.4)	47.6 (6.8)	48.1 (7.1)	0.41
Smoking status, %								
Current	35.2	35.6	35.2	0.95	39.9	27.5	32.7	0.14
Past	24.8	26.3	26.9		24.4	32.6	29.6	
Never	39.4	37.1	37.2		35.2	39.9	36.7	
Missing	0.6	1.0	0.8		0.5	0.0	1.0	
Physical activity, %								
Yes	63.1	55.9	55.0	0.03	61.7	56.0	51.3	0.16
No	35.7	42.2	43.3		37.3	43.0	45.7	
Missing	1.2	1.9	1.7		1.0	1.0	3.0	
Drinking habit, %								
Overdrinking	11.5	10.8	12.9	0.48	13.5	15.5	14.1	0.85
Family history of diabetes, %								
Yes	12.3	11.6	12.4	0.87	16.6	15.0	20.6	0.32
Body mass index, kg/m^2^	21.4 (1.9)	22.2 (1.7)	22.7 (1.5)	<0.001	26.2 (1.1)	26.5 (1.5)	27.1 (1.8)	<0.001
Triglycerides, mg/dL^b^	80.0 (60.7, 106.0)	103.0 (80.0, 143.0)	143.0 (99.0, 206.0)	<0.001	110.0 (85.0, 148.5)	136.0 (96.5, 187.0)	185.0 (131.0, 259.0)	<0.001
Adiponectin, µg/mL^b^	7.6 (5.7, 9.7)	6.4 (5.0, 8.5)	5.7 (4.2, 7.4)	<0.001	6.0 (4.8, 7.9)	5.1 (4.1, 6.9)	4.6 (3.7, 6.1)	<0.001
Alanine transaminase, IU/L^b^	18.0 (14.0, 23.0)	20.0 (16.0, 27.0)	25.0 (18.0, 36.0)	<0.001	24.0 (17.5, 33.0)	29.0 (22.0, 40.0)	34.0 (26.0, 55.0)	<0.001
Gamma-glutamyl transpeptidase, IU/L^b^	27.0 (19.0, 46.0)	32.0 (22.0, 51.0)	41.0 (26.0, 70.0)	<0.001	39.0 (26.0, 58.5)	48.0 (31.5, 75.0)	56.0 (36.0, 88.0)	<0.001
Insulin, µU/L^b^	3.0 (3.0, 5.0)	5.0 (4.0, 7.0)	8.0 (7.0, 12.0)	<0.001	6.0 (4.0, 7.0)	8.0 (7.0, 10.0)	11.0 (9.0, 15.0)	<0.001
Free fatty acid, mEq/L	0.6 (0.2)	0.7 (0.3)	0.9 (0.4)	<0.001	0.6 (0.2)	0.8 (0.3)	1.1 (0.4)	<0.001
Blood glucose, mg/dL	89.6 (8.6)	92.5 (8.7)	95.9 (9.8)	<0.001	92.3 (8.0)	94.0 (8.4)	95.6 (10.7)	0.002
HbA1c, %	4.9 (0.3)	4.8 (0.4)	4.9 (0.4)	0.14	4.9 (0.4)	5.0 (0.4)	5.1 (0.3)	0.002
Hypertension medication, %	4.5	5.5	6	0.48	5.8	10.9	11.1	0.13
Hyperlipidemia medication, %	1.6	2.3	3.1	0.18	3.2	6.3	5.1	0.38

Adipo-IR, adipocyte insulin resistance index; HbA1c, glycosylated hemoglobin.

Data are shown as mean (standard deviation) for continuous variable and percentage (%) for categorical variable or indicated otherwise.

^a^Sex and BMI category-specific Adipo-IR tertile.

^b^Median and interquartile range. *P* values were determined by analysis of variance and χ^2^ tests for normally distributed continuous and categorical data, respectively. For nonnormally distributed continuous data, *P* values were determined by Kruskal-Wallis test.

**Table 2.** Baseline characteristics according to Adipo-IR tertiles stratified by BMI-category in women, Aichi Worker’s Cohort study, 2002 (*n* = 756)

	Normal Weight				Overweight/Obesity			
	
	Adipo-IR tertile^a^			*P*	Adipo-IR tertile^a^			*P*
	
	T1	T2	T3		T1	T2	T3	
Number of subjects	220	223	225		29	29	30	
Median Adipo-IR (range)	13.7 (2.6–19.8)	26.5 (20.0–33.9)	46.2 (34.2–160.9)		23.1 (11.0–33.1)	46.3 (33.1–60.1)	83.7 (61.8–225.4)	
Age, years	46.5 (6.9)	46.3 (6.7)	45.4 (7.1)	0.18	48.3 (6.8)	45.2 (5.9)	46.3 (6.2)	0.16
Smoking status, %								
Current	7.7	4.5	7.1	0.54	0.0	10.3	23.3	0.01
Past	3.6	5.8	4.0		3.4	10.3	0.0	
Never	88.2	88.3	87.1		96.6	79.3	76.7	
Missing	0.5	1.3	1.8		0.0	0.0	0.0	
Physical activity, %								
Yes	46.8	40.4	44.9	0.006	37.9	55.2	20.0	0.04
No	50.0	59.2	55.1		62.1	41.4	76.7	
Missing	3.2	0.4	0.0		0.0	3.4	3.3	
Drinking habit, %								
Overdrinking	5.9	6.7	8.4	0.58	3.4	3.4	3.3	0.99
Family history of diabetes, %								
Yes	19.5	17.9	16.9	0.77	24.1	31.0	26.7	0.87
Body mass index, kg/m^2^	20.4 (1.8)	21.1 (1.8)	21.6 (2.0)	<0.001	27.9 (5.7)	26.9 (1.6)	27.8 (2.1)	0.55
Triglycerides, mg/dL^b^	61.0 (49.0, 87.0)	68.0 (53.0, 95.0)	82.0 (60.0, 121.0)	<0.001	82.0 (64.5, 116.5)	90.0 (73.5, 116.0)	129.5 (95.0, 191.2)	<0.001
Adiponectin, µg/mL^b^	11.3 (8.4, 15.5)	10.5 (8.3, 13.1)	8.9 (6.5, 12.0)	<0.001	7.6 (5.9, 11.5)	7.6 (5.2, 9.5)	6.0 (4.1, 7.1)	0.02
Alanine transaminase, IU/L^b^	13.0 (10.0, 16.0)	13.0 (11.0, 17.0)	15.0 (11.0, 20.0)	<0.001	17.0 (13.0, 25.5)	19.0 (14.5, 29.0)	26.5 (15.7, 44.0)	0.01
Gamma-glutamyl transpeptidase, IU/L^b^	14.0 (12.0, 19.0)	16.0 (13.0, 23.0)	18.0 (14.0, 28.5)	<0.001	22.0 (17.0, 31.5)	23.0 (15.0, 38.5)	31.0 (22.7, 48.7)	0.05
Insulin, µU/L^b^	3.0 (3.0, 5.0)	5.0 (4.0, 7.0)	8.0 (6.5, 11.0)	<0.001	6.0 (4.0, 9.5)	9.0 (7.0, 10.5)	15.0 (11.7, 24.7)	<0.001
Free fatty acid, mEq/L	0.5 (0.2)	0.7 (0.2)	0.9 (0.4)	<0.001	0.5 (0.2)	0.8 (0.2)	0.9 (0.5)	<0.001
Blood glucose, mg/dL	85.6 (7.1)	89.0 (8.4)	92.9 (10.2)	<0.001	90.6 (8.7)	93.2 (8.4)	94.8 (11.7)	0.25
HbA1c, %	4.8 (0.3)	4.8 (0.3)	5.0 (0.5)	0.06	5.0 (0.4)	4.7 (0.8)	4.9 (0.3)	0.64
Hypertension medication, %	2.3	2.7	3.6	0.77	7.1	7.1	6.7	0.99
Hyperlipidemia medication, %	1.8	0.9	2.7	0.39	0.0	0.0	6.7	0.32

Adipo-IR, adipocyte insulin resistance index; HbA1c, glycosylated hemoglobin.

Data are shown as mean (standard deviation) for continuous variable and percentage (%) for categorical variable or indicated otherwise.

^a^Sex and BMI category-specific Adipo-IR tertile.

^b^Median and interquartile range. *P* values were determined by analysis of variance and χ^2^ tests for normally distributed continuous and categorical data, respectively. For nonnormally distributed continuous data, *P* values were determined by Kruskal-Wallis test.

During 17 (median 14.6) years of follow-up, a total of 365 T2DM cases were identified (men: 296; women: 69). The incidence rates of T2DM per 1,000 person-years across Adipo-IR tertiles (T1, T2, and T3) were 6.2, 7.0, and 10.5 for normal-weight men and 9.5, 12.4, and 29.4 for overweight/obese men. The corresponding incidence rates for women were 5.9, 4.3, and 8.0 in normal-weight women and 9.3, 28.3, and 46.4 in overweight/obese women (Table 3).

**Table 3.** Hazard ratios and 95% confidence intervals of type 2 diabetes according to Adipo-IR tertile stratified by BMI category in men and women, Aichi Workers’ Cohort Study, 2002–2019 (*n* = 3,257)

	Normal weight				Overweight/Obesity			
	
	Adipo-IR tertile^a^			Total/continuous^b^	Adipo-IR tertile^a^			Total/continuous^b^
	
	T1	T2	T3		T1	T2	T3	
**Men**								
*n*/*N*	50/642	54/623	82/651	186/1,916	22/193	28/193	60/199	110/585
Incidence rate^c^	6.2	7.0	10.5	7.9	9.5	12.4	29.4	16.6
Age-adjusted model	1	1.12 (0.76–1.64)	1.67 (1.17–2.37)	1.22 (1.07–1.38)	1	1.32 (0.75–2.31)	3.14 (1.92–5.13)	1.31 (1.16–1.47)
Multivariable model	1	1.11 (0.75–1.64)	1.62 (1.12–2.34)	1.20 (1.06–1.37)	1	1.37 (0.77–2.41)	2.94 (1.76–4.90)	1.25 (1.10–1.42)

**Women**								
*n*/*N*	15/220	11/223	20/225	46/668	3/29	8/29	12/30	23/88
Incidence rate^c^	5.9	4.3	8.0	6.0	9.3	28.3	46.4	26.6
Age-adjusted model	1	0.74 (0.34–1.62)	1.48 (0.76–2.90)	1.29 (0.97–1.70)	1	3.20 (0.82–12.49)	5.06 (1.42–18.05)	1.53 (1.25–1.87)
Multivariable model	1	0.63 (0.28–1.42)	1.16 (0.57–2.36)	1.18 (0.87–1.60)	1	2.91 (0.70–12.13)	4.24 (1.08–16.61)	1.66 (1.29–2.12)

Adipo-IR, adipocyte insulin resistance index; *n*, number of cases; *N*, number of participants.

Multivariable model includes age, body mass index, smoking status, physical activity, family history of diabetes and drinking habit.

^a^Sex and body mass index-category-specific Adipo-IR tertile.

^b^Continuous analyses were performed using a one standard deviation increase in Adipo-IR.

^c^Incidence rate is expressed as per 1,000 person-years.

As the proportional hazard assumption was not violated in Cox models stratified by sex and BMI category based on the Schoenfeld residuals, we evaluated the multivariable-adjusted associations of Adipo-IR with incident T2DM. In the multivariable model, the HRs of T3 vs T1 were 1.62 (95% CI, 1.12–2.34) in normal-weight men and 1.16 (95% CI, 0.57–2.36) in normal-weight women. The corresponding values were 2.94 (95% CI, 1.76–4.90) in overweight/obese men and 4.24 (95% CI, 1.08–16.6) in overweight/obese women. Although the associations seem stronger in overweight/obese men and women, the interaction between Adipo-IR and BMI-category was not significant (*P* = 0.28 and 0.27 in men and women, respectively). Furthermore, the association of Adipo-IR with T2DM risk appears to be stronger in overweight/obese women than in overweight/obese men (*P* for interaction between Adipo-IR and sex = 0.04). Analyses using Adipo-IR as a continuous variable yielded similar findings: each 1-SD increase in Adipo-IR was associated with a 25% higher risk in overweight/obese men (HR 1.25; 95% CI, 1.10–1.42) and 66% higher risk in overweight/obese women (HR 1.66; 95% CI, 1.29–2.12). While HOMA-IR showed a similar association in overweight/obese men (HR per 1-SD, 1.26; 95% CI, 1.12–1.41), it was not statistically significantly associated with T2DM risk in women (HR 0.98; 95% CI, 0.76–1.28).

Similar findings were obtained from sensitivity analyses. Censoring the participants at the age of 60 years did not change the association in both overweight/obese men in the multivariable model (HR for T3 vs T1, 2.88; 95% CI, 1.60–5.20; HR per 1-SD, 1.22; 95% CI, 1.06–1.41) and women (HR for T3 vs T1, 4.37; 95% CI, 1.05–18.12; HR per 1-SD, 1.70; 95% CI, 1.29–2.24) (eTable 2). Similarly, results did not materially change when outliers were not excluded (multivariable model HR for T3 vs T1, 2.85; 95% CI, 1.73–4.72 in men and 5.78; 95% CI, 1.53–21.9 in women) or a more stringent exclusion of outliers (≥95th percentile) was applied (multivariable model HR for T3 vs T1, 2.73; 95% CI, 1.57–4.76 in men and 2.55; 95% CI, 0.55–11.84 in women). Similar associations were observed when Adipo-IR was treated as a log-transformed continuous variable (HRs per one-unit increment of log-transformed Adipo-IR, 2.02; 95% CI, 1.48–2.75 in men and 4.03; 95% CI, 1.71–9.51 in women). Furthermore, a consistent association was observed when using sex-specific Adipo-IR tertile cutoff values without stratifying by BMI category in both men (HR for T3 vs T1, 2.07; 95% CI, 1.50–2.86) and in women (HR for T3 vs T1, 2.12; 95% CI, 1.17–3.83) (eTable 3).

## DISCUSSION

The main findings of the present study are that baseline variation of Adipo-IR was associated with differences in the risk of developing T2DM especially in overweight/obese men and women, and that the association appears to be stronger in overweight/obese women than in overweight/obese men. Positive association was also found in normal-weight men but not in normal-weight women. These associations were independent of potential confounding factors, including age, BMI, smoking status, physical activity, alcohol drinking, and family history of diabetes.

The present result is generally consistent with a shorter follow-up study of individuals at high risk of T2DM^11^^,^^12^ and several cross-sectional studies.^8^^,^^20^^–^^24^ We extended these findings to an apparently healthy Asian population with a much larger sample size, which enabled sex- and BMI-category-specific analyses. We also found that higher Adipo-IR tertiles were strongly associated with TG, adiponectin, ALT, and GGT across both BMI categories in both sexes, and with BMI and blood glucose level in normal-weight men and women, as well as in overweight/obese men. To note, while HOMA-IR showed a significant association with T2DM similar to Adipo-IR in men, it was not related to T2DM risk in overweight/obese women. This may be a clinical implication of the present study, suggesting that adipocyte insulin resistance may be more pathogenetically related to the development of T2DM in overweight/obese women than systemic or hepatic insulin resistance.

A significant but weaker association between Adipo-IR and T2DM incidence was observed among normal-weight men, but not in normal-weight women, in contrast to the seemingly stronger association in overweight/obese individuals in the present study. This finding—that the degree of adipose tissue insulin resistance in the absence of obesity was related to T2DM incidence—may reflect the presence of individuals with a lower BMI threshold for adipose tissue expandability^25^ (ie, interindividual differences in adipocyte function), particularly in men. Additionally, the contribution of impaired insulin secretion may be greater in normal-weight individuals.^26^ Nevertheless, we found significant associations between Adipo-IR and baseline clinical variables, including glucose in normal-weight individuals, in line with previous studies.^21^^,^^23^

It has been reported that both total and visceral obesity, key risk factors for the development of T2DM, were strongly associated with Adipo-IR.^20^^,^^24^ An increase in adiposity is accompanied by adipocyte hypertrophy. Hypertrophic adipocytes become resistant to the action of insulin through inflammatory mechanisms mediated by pro-inflammatory adipocytokines. Surplus FFA leads to elevated circulating FFA levels,^27^ which in turn promote ectopic fat accumulation, particularly in the liver and muscle. This would causally be related to insulin resistance in the liver and skeletal muscle,^27^^–^^29^ as well as to metabolic syndrome,^22^ and T2DM.^9^^,^^10^^,^^24^ In the present study, we demonstrated an association between Adipo-IR and T2DM, independent of confounding factors. The above-mentioned causal pathway could be examined epidemiologically in the future through causal mediation analysis, using additional information on liver marker levels collected during follow-up.

In the present study, the degree of positive association of Adipo-IR with T2DM was stronger among overweight/obese women compared to overweight/obese men. Although the reason for the sex-related differences in the magnitude of the association remains to be elucidated in future studies, it may be partially consistent with a previous finding of a stronger association of Adipo-IR with nonalcoholic fatty liver disease in hyperlipidemic women.^30^ Excess adiposity in women, a state with high adipose-tissue-derived estrogen without an increase of progesterone,^31^ resembled that observed in unopposed estrogen use, which had also been associated with T2DM risk.^32^ We speculated that the women’s greater susceptibility to excess adiposity for the deterioration of glucose metabolism was due to increased ectopic fat deposition resulting from high FFA flux.^33^ Another clinically relevant finding of the present study was that Adipo-IR, not HOMA-IR was predictive of future development of T2DM in overweight/obese women. Future studies are needed to examine whether incorporating the Adipo-IR index into established risk factors can meaningfully improve the prediction model.

Although the present study had many strengths described above, it has limitations that should be discussed. First, a gold standard method for measuring adipose tissue insulin sensitivity, multistep pancreatic clamp method, was not performed in the present study. However, that method used tracer dilution techniques to estimate lipolysis fluxes during continuous intravenous insulin infusion,^34^ which would be inappropriate for large-sample observational studies. Nevertheless, Adipo-IR showed a strong correlation (*r* = 0.86) with 50% suppression of lipolysis (IC_50_) determined by multistep pancreatic clamp technique.^7^^,^^35^ Second, Adipo-IR was measured only at baseline. The present study should be regarded as a long-term association of baseline Adipo-IR with the development of T2DM. Since it was not known how Adipo-IR would change during the long-term course from normal to T2DM onset, future epidemiological studies should also be performed to incorporate multiple Adipo-IR measurements during the follow-up. Third, we mainly relied on the annual health check-up results (85% of total cases), approximately 70% of which were based on FBG measurement for the ascertainment of T2DM. Cases that met FBG criteria and those that met HbA1c may have different characteristics.^36^^,^^37^ Further studies examining possible differences in the associations by T2DM ascertainment method, obtaining both FBG and HbA1c, should be conducted. Fourth, self-antibodies, such as glutamic acid decarboxylase autoantibodies (GADA) or islet-specific autoantibodies (ISA), were not obtained in the present study for defining T2DM. Therefore, there was a possibility that slowly progressive insulin-dependent diabetes mellitus was included in our T2DM cases. Even though the proportion was reportedly small,^38^ which was also confirmed through our detailed surveys of the physicians following the self-reported surveys, this might have somehow contributed to our insignificant findings in the normal-weight stratum. Fifth, approximately 70% of the participants reached the standard retirement age of 60 years during follow-up, and about half of them were subsequently followed-up via questionnaire, based on prior consent. We considered this loss to follow-up due to retirement (approximately 20%) to be unrelated to participants’ health status and unlikely to have biased the observed associations, as the consent was obtained well in advance. Nevertheless, we conducted a sensitivity analysis in which all participants were censored at age 60 years and found that this did not materially alter the results. Sixth, in the present study, total FFA level was used for the analysis. Since adipose tissue insulin resistance may have different effects on the levels of different types of FFA, such as saturated, mono-unsaturated, and poly-unsaturated FFA, and also their pathophysiological significance in relation to T2DM development may not be the same,^39^ future studies incorporating the types of FFA for the assessment of Adipo-IR are warranted. Seventh, because of the small sample sizes of women, some analyses were underpowered to provide definite evidence. Finally, the population we studied was limited to middle-aged Japanese workers. Further investigations should be performed including subjects of all age groups, occupations, and ethnicities.

In conclusion, the present study suggested that higher tertile of Adipo-IR was strongly associated with increased incidence of T2DM in overweight/obese people compared to lower tertile irrespective of age, BMI, smoking status, physical activity, alcohol drinking, and family history of diabetes.

## SUPPLEMENTARY MATERIAL

eTable 1. Pearson correlation coefficients of Adipo-IR with age, and anthropometric and clinical variables stratified by BMI-category in men and women, Aichi Workers’ Cohort Study, 2002 (n = 3,257)

eTable 2. Hazard ratios and 95% confidence intervals of type 2 diabetes according to Adipo-IR tertile stratified by BMI category in men and women after censoring the retiree participants, Aichi Workers’ Cohort Study, 2002–2019 (n = 3,154)

eTable 3. Hazard ratios and 95% confidence intervals of type 2 diabetes according to sex-specific Adipo-IR tertile in men and women, Aichi Workers’ Cohort Study, 2002–2019 (n = 3,257)

eFigure 1. Flow chart of selection of study population, Aichi Workers’ Cohort Study, 2002. Adipo-IR, adipocyte insulin resistance index.

eFigure 2. Pathophysiological model of Adipose tissue insulin resistance and T2DM. T2DM, type 2 diabetes mellitus; TG, triglycerides.
