# Supplementary material for: Association of Adipose Tissue Insulin Resistance With Risk of Diabetes Incidence in Middle-aged Japanese Workers According to BMI States: 17 Years of Follow-up of the Aichi Worker’s Cohort Study
Source: J Epidemiol. 2026 Jan 5;36(1):44–50. doi: 10.2188/jea.JE20250025 (PMC12698324; doi:10.2188/jea.JE20250025)
Supplement: Supplementary file 1 [file je-36-044-s001.pdf]

eTable 1. Pearson correlation coefficients of Adipo-IR with age, and anthropometric and clinical variables stratified by BMI-category in men and women, Aichi Workers' Cohort Study, 2002 (n=3,257)

Variables	Normal weight	Overweight/obesity
**Men**		
Age, years	0.02	0.001
Body mass index, kg/m^2	0.22^{***}	0.27^{***}
Triglycerides, mg/dL	0.40^{***}	0.45^{***}
Adiponectin, μg/mL	-0.22^{***}	-0.22^{***}
Alanine transaminase, IU/L	0.27^{***}	0.29^{***}
Gamma-glutamyl transpeptidase, IU/L	0.14^{***}	0.13^{***}
Blood glucose, mg/dL	0.27^{***}	0.12^{***}
HbA1c, mg/dL	0.06	0.28^{***}
**Women**		
Age, years	-0.05	-0.16
Body mass index, kg/m^2	0.23^{***}	-0.04
Triglycerides, mg/dL	0.30^{***}	0.38^{***}
Adiponectin, μg/mL	-0.20^{***}	-0.36^{***}
Alanine transaminase, IU/L	0.11^{***}	0.42^{***}
Gamma-glutamyl transpeptidase, IU/L	0.14^{***}	0.15
Blood glucose, mg/dL	0.33^{***}	0.07
HbA1c, mg/dL	0.13	-0.17

Adipo-IR, adipocyte insulin resistance index; BMI, body mass index.

*** P<0.01.

eTable 2. Hazard ratios and 95% confidence intervals of type 2 diabetes according to Adipo-IR tertile stratified by BMI category in men and women after censoring the retiree participants, Aichi Workers' Cohort Study, 2002–2019 (n=3,154)

	Normal weight				Overweight/Obesity			
	Adipo-IR tertile[a]			Total/ continuous[b]	Adipo-IR tertile[a]			Total/ continuous[b]
	T1	T2	T3		T1	T2	T3	
**Men**								
n/N	35/620	44/602	59/626	138/1,848	16/188	21/184	49/191	86/563
Incidence rate[c]	5.4	7.3	9.4	7.3	8.9	11.8	28.8	16.3
Age-adjusted model	1	1.32 (0.85–2.06)	1.67 (1.10–2.54)	1.23 (1.07–1.42)	1	1.35 (0.70–2.59)	3.33 (1.89–5.86)	1.29 (1.13–1.47)
Multivariable model	1	1.30 (0.83–2.04)	1.58 (1.02–2.44)	1.21 (1.05–1.41)	1	1.32 (0.68–2.55)	2.88 (1.60–5.20)	1.22 (1.06–1.41)
**Women**								
n/N	13/217	11/218	19/222	43/657	3/28	7/29	10/29	20/86
Incidence rate[c]	6.0	4.9	8.5	6.4	11.6	25.0	46.7	26.5
Age-adjusted model	1	0.83 (0.37–1.86)	1.57 (0.77–3.19)	1.28 (0.96–1.70)	1	2.21 (0.56–8.72)	4.14 (1.14–15.09)	1.48 (1.19–1.84)
Multivariable model	1	0.70 (0.31–1.63)	1.23 (0.58–2.60)	1.17 (0.86–1.58)	1	2.11 (0.50–9.00)	4.37 (1.05–18.12)	1.70 (1.29–2.24)

Adipo-IR, adipocyte insulin resistance index; n, number of cases; N, number of participants.
Multivariable model includes age, body mass index, smoking status, physical activity, family history of diabetes and drinking habit.
[a]Sex and BMI category-specific Adipo-IR tertile.
[b]Continuous analyses were performed using a one standard deviation increase in Adipo-IR.
[c]Incidence rate is expressed as per 1,000 person-years.

eTable 3. Hazard ratios and 95% confidence intervals of type 2 diabetes according to sex-specific Adipo-IR tertile in men and women, Aichi Workers' Cohort Study, 2002–2019 (n=3,257)

	**Adipo-IR tertile**			**Total/ continuous[a]**
	**T1**	**T2**	**T3**	
**Total[b]**				
n/N	76/1,077	106/1,077	183/1,103	365/3,257
Incidence rate[c]	5.7	8.2	14.6	9.5
Age-adjusted model	1	1.45 (1.08–1.95)	2.57 (1.97–3.36)	1.36 (1.27–1.46)
Multivariable model	1	1.34 (0.99–1.80)	2.04 (1.55–2.68)	1.25 (1.17–1.35)
**Men[d]**				
n/N	59/827	91/828	146/846	296/2,501
Incidence rate[c]	5.7	9.1	14.9	9.8
Age-adjusted model	1	1.60 (1.15–2.22)	2.61 (1.92–3.53)	1.32 (1.22–1.43)
Multivariable model	1	1.44 (1.03–2.01)	2.07 (1.50–2.86)	1.22 (1.11–1.33)
**Women[d]**				
n/N	17/250	16/251	36/255	69/756
Incidence rate[c]	5.9	5.5	13.4	8.1
Age-adjusted model	1	0.96 (0.48–1.89)	2.44 (1.36–4.36)	1.61 (1.40–1.85)
Multivariable model	1	1.01 (0.50–2.03)	2.12 (1.17–3.83)	1.51 (1.29–1.77)

Adipo-IR, adipocyte insulin resistance index; n, number of cases; N, number of participants.
Multivariable model includes age, body mass index, smoking status, physical activity, family history of diabetes and drinking habit.
[a]Continuous analyses were performed using a one standard deviation increase in Adipo-IR.
[b]Tertile analyses were performed using Adipo-IR tertiles defined in the total population.
[c]Incidence rate is expressed as per 1,000 person-years.
[d]Tertile analyses were performed using sex-specific Adipo-IR tertiles.

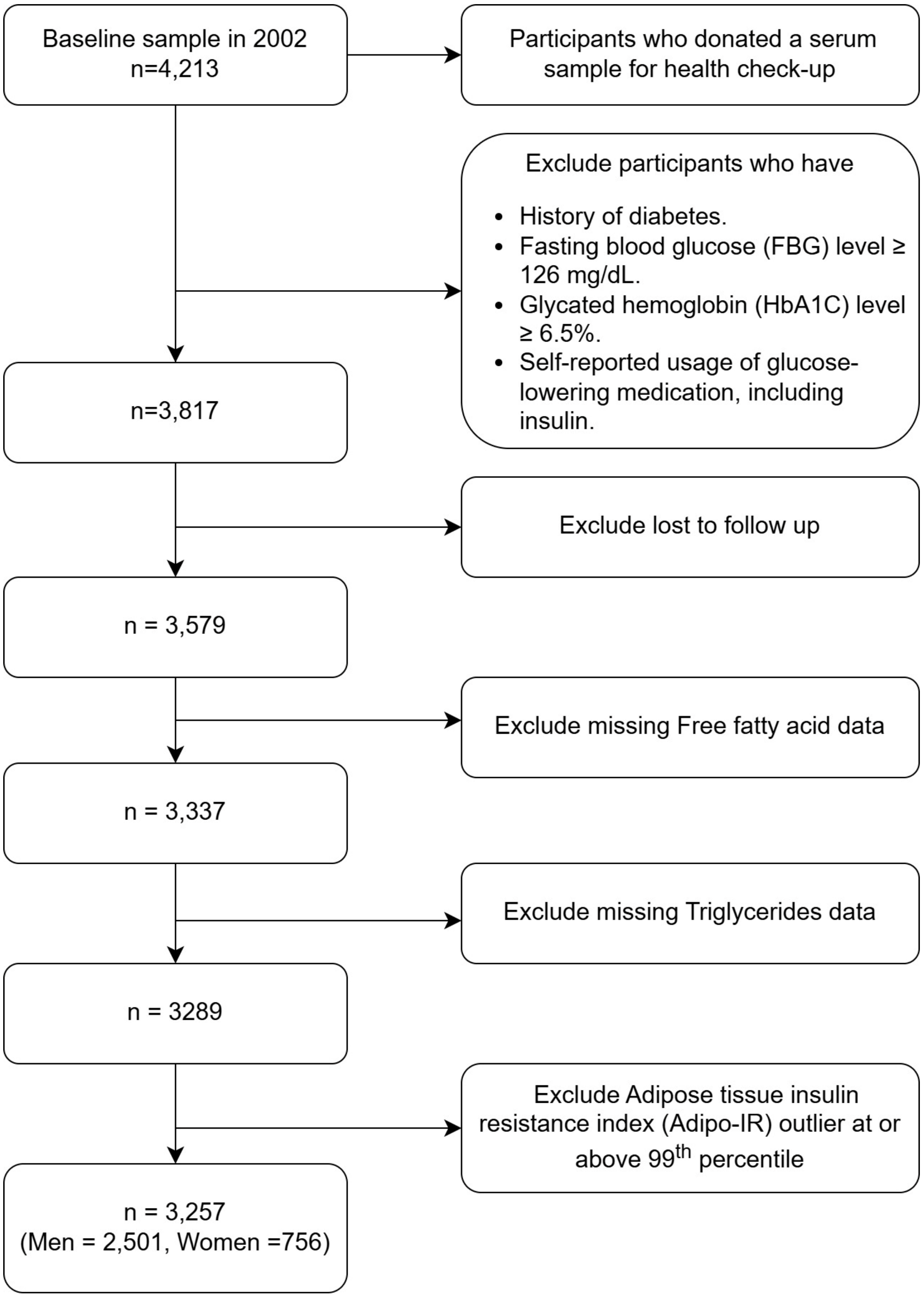

eFigure 1. Flow chart of selection of study population, Aichi Workers' Cohort Study, 2002. Adipo-IR, adipocyte insulin resistance index.

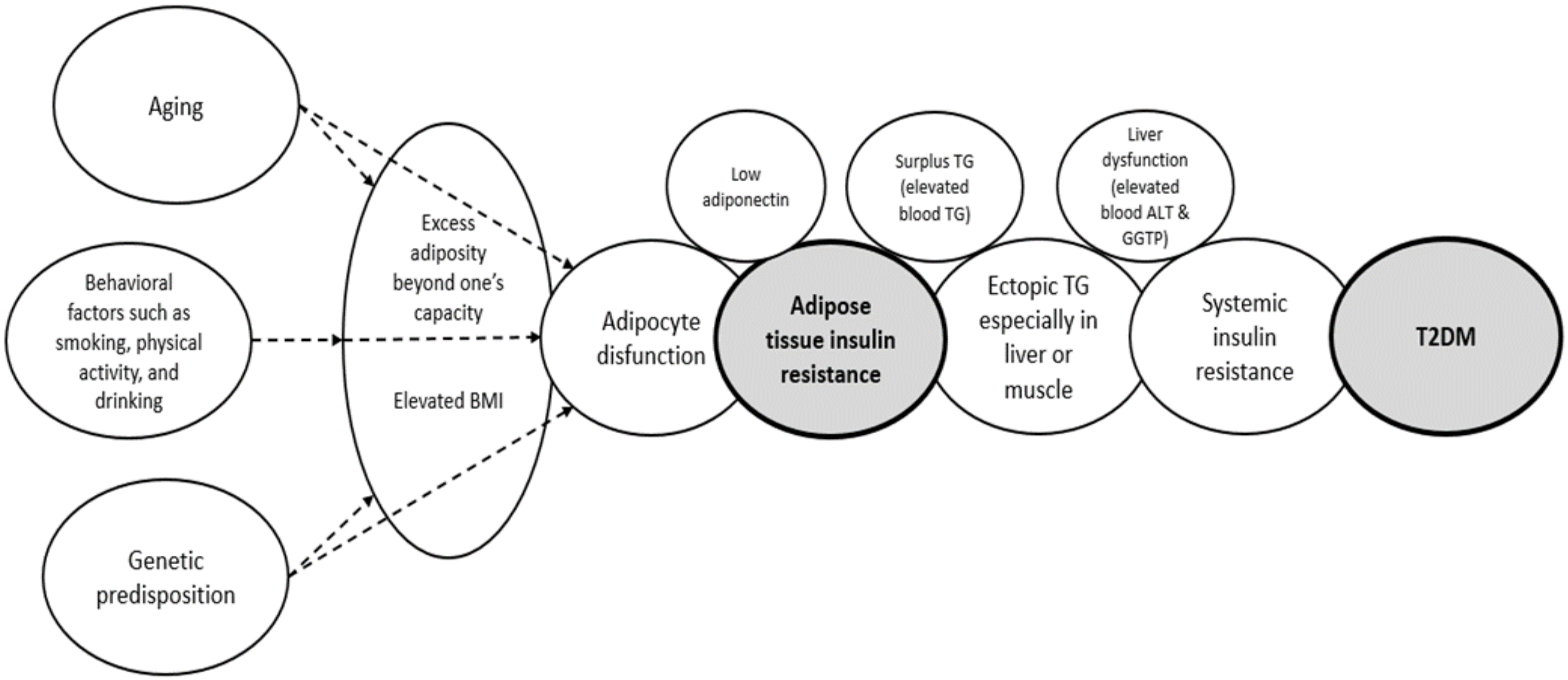

eFigure 2. Pathophysiological model of Adipose tissue insulin resistance and T2DM. T2DM, type 2 diabetes mellitus; TG, triglycerides.
